# Correction to: m6A mRNA methylation initiated by METTL3 directly promotes YAP translation and increases YAP activity by regulating the MALAT1-miR-1914-3p-YAP axis to induce NSCLC drug resistance and metastasis

**DOI:** 10.1186/s13045-020-00942-x

**Published:** 2020-08-03

**Authors:** Dan Jin, Jiwei Guo, Yan Wu, Jing Du, Lijuan Yang, Xiaohong Wang, Weihua Di, Baoguang Hu, Jiajia An, Lingqun Kong, Lei Pan, Guoming Su

**Affiliations:** 1 Clinical Medical Laboratory, Binzhou Medical University Hospital, Binzhou, 256603 People’s Republic of China; 2 Cancer research institute, Binzhou Medical University Hospital, Binzhou, 256603 People’s Republic of China; 3 Department of Thyroid and Breast Surgery, Binzhou Medical University Hospital, Binzhou, 256603 People’s Republic of China; 4 Department of Pain, Binzhou Medical University Hospital, Binzhou, 256603 People’s Republic of China; 5 Department of Gastrointestinal Surgery, Binzhou Medical University Hospital, Binzhou, 256603 People’s Republic of China; 6 Department of Clinical Laboratory, Binzhou Medical University Hospital, Binzhou, 256603 People’s Republic of China; 7 Department of Hepatobiliary Surgery, Binzhou Medical University Hospital, Binzhou, 256603 People’s Republic of China; 8 Department of Respiratory and Critical Care Medicine, Binzhou Medical University Hospital, Binzhou, 256603 People’s Republic of China; 9 Department of Nursing, Binzhou Polytechnic University, Binzhou, 256603 People’s Republic of China

**Correction to: J Hematol Oncol 12:135 (2019)**

**https://doi.org/10.1186/s13045-019-0830-6**

The original article [1] contains errors in Fig. 2h, Fig. 2n and Fig. 6k:

1. In Fig. 2h, the protein band of YTHDF3 was mistakenly duplicated into the protein band of Cyr61.
2. In Fig. 2n, the image of Control+Vector treatment group was mistakenly duplicated into the siMETTL3 + YTHDF3 treatment group, and the image of METTL3 + siYTHDF3 treatment group was unintentionally duplicated into the METTL3 + YTHDF3 treatment group which were determined by transwell assay.
3. In Fig. 6k, the IHC image of ABCG2 was unintentionally duplicated onto the IHC image of ERCC1.

**Fig. 2:**
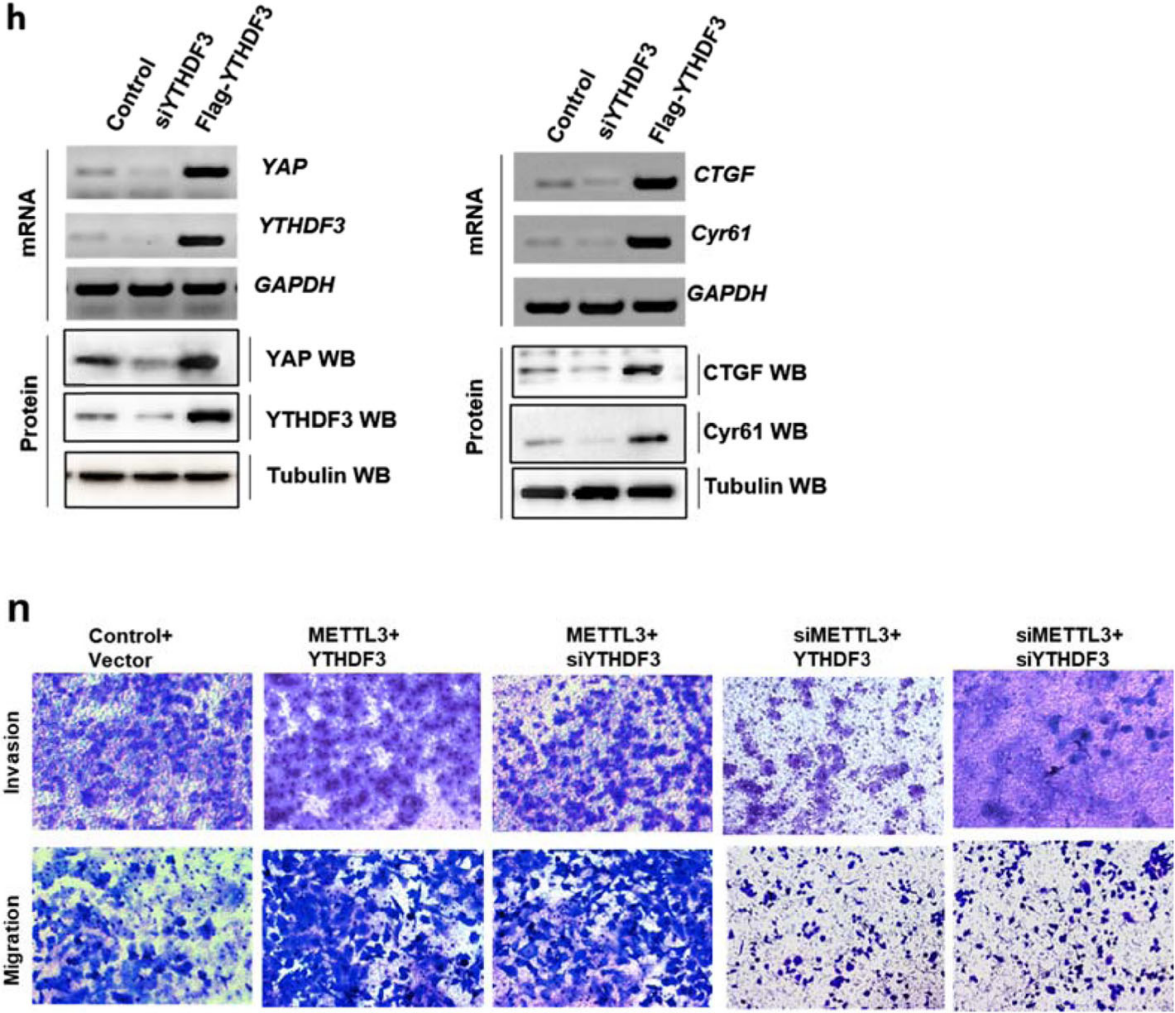
YTHDF3 recognizes m^6^A modification and promotes cellular growth and migration via YAP upregulation. **h** The expressions of YAP and its target genes, CTGF and Cyr61, were analyzed by RT-PCR and western blot. **n** The cellular invasion and migration growths were analyzed by transwell assay. Results were presented as mean ± SD of three independent experiments. *P < 0.05 or **P < 0.01 indicates a significant difference between the indicated groups. NS, not significant

**Fig. 6:**
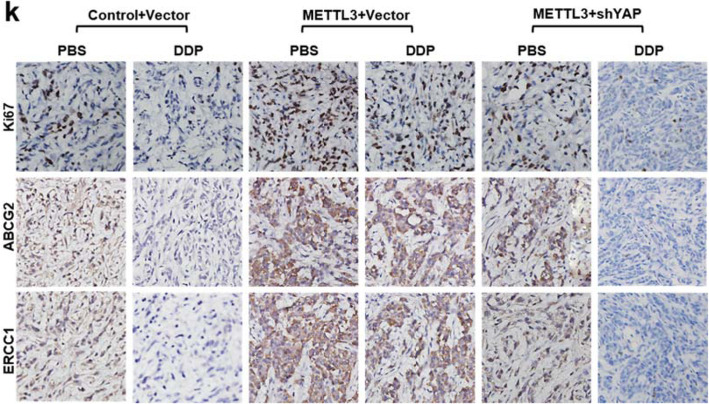
The reduction of YAP m6A modification inhibits tumor growth and enhances DDP sensitivity in vivo. **k** The tumor nodules from control^vector^, METTL3^Vector^ and METTL3^shYap^ groups were treated with PBS or DDP every three days. The protein expression levels of Ki67, ABCG2 and ERCC1were analyzed immunohistochemical staining assays (*n* = 5). Results were presented as mean ± SD three independent experiments. **P < 0.01 indicates a significant difference between the indicated groups. NS, not significant

These errors were mistakenly introduced when organising the results; however, these errors have no bearing on the work’s scientific conclusions as the statistical results are based on the correct pictures.

The authors would like to note the correct versions of each of the above-noted sub-figures ahead. The only changes are to the panels of Fig. 2h, Fig. 2n, and Fig. 6k, and the rest of the figures are identical to the published version; furthermore, no other errors were found after repeated checking of the published data.

The authors apologise to the Editor of Journal of Hematology & Oncology and to the readership for any inconvenience caused.
